# Energy Dissipation in Graphene Mechanical Resonators with and without Free Edges

**DOI:** 10.3390/mi7090158

**Published:** 2016-09-05

**Authors:** Makoto Takamura, Hajime Okamoto, Kazuaki Furukawa, Hiroshi Yamaguchi, Hiroki Hibino

**Affiliations:** NTT Basic Research Laboratories, NTT Corporation, 3-1 Morinosato Wakamiya, Atsugi, Kanagawa 243-0198, Japan; okamoto.hajime@lab.ntt.co.jp (H.O.); yamaguchi.hiroshi@lab.ntt.co.jp (H.Y.)

**Keywords:** graphene, nanoelectromechanical systems (NEMS), microelectromechanical systems (MEMS), hydrogen intercalation, electrochemical etching, energy dissipation

## Abstract

Graphene-based nanoelectromechanical systems (NEMS) have high future potential to realize sensitive mass and force sensors owing to graphene’s low mass density and exceptional mechanical properties. One of the important remaining issues in this field is how to achieve mechanical resonators with a high quality factor (*Q*). Energy dissipation in resonators decreases *Q*, and suppressing it is the key to realizing sensitive sensors. In this article, we review our recent work on energy dissipation in doubly-clamped and circular drumhead graphene resonators. We examined the temperature (*T*) dependence of the inverse of a quality factor ($Q^{-1}$) to reveal what the dominant dissipation mechanism is. Our doubly-clamped trilayer resonators show a characteristic $Q^{-1}$-*T* curve similar to that observed in monolayer resonators: $Q^{-1}$ ∝ $T^{2}$ above ∼100 K and ∝ $T^{0.3}$ below ∼100 K. By comparing our results with previous experimental and theoretical results, we determine that the $T^{2}$ and $T^{0.3}$ dependences can be attributed to tensile strain induced by clamping metals and vibrations at the free edges in doubly-clamped resonators, respectively. The $Q^{-1}$-*T* curve in our circular drumhead resonators indicates that removing free edges and clamping metal suppresses energy dissipation in the resonators, resulting in a linear *T* dependence of $Q^{-1}$ in a wide temperature range.

## 1. Introduction

Graphene resonators are well suited for use in nanoelectromechanical systems (NEMS) owing to graphene’s low mass density and exceptional mechanical properties, such as its high Young’s modulus. Its low mass density is especially useful for designing ultrasensitive mass and force sensors, because the lighter mass of a resonator improves the resolution of mass or force detection. For example, the minimum detectable mass against a background of white noise is given by δm ∼ 2($m_{\mathrm{eff}}$/*Q*)$10^{\left(\mathrm{DR}/20\right)}$ [1], where $m_{\mathrm{eff}}$ is the effective mass of the resonator, *Q* the quality factor and DR the dynamic range. To maximize the mass and force sensitivity, high *Q* = *f*/Δf is required in addition to the small $m_{\mathrm{eff}}$ resulting from the low mass density of graphene, where *f* is the resonance frequency and Δf is the full width at half maximum of the Lorentzian amplitude response peak. A recent study has reported that suspended graphene resonators show high *Q* > $10^{4}$ at cryogenic temperatures, yielding the highly sensitive mass detection on the order of $10^{-21}$ g [2]. However, it has also been reported that *Q* drastically decreases with increasing temperature, and *Q* at room temperature is very low (typically, ∼$10^{2}$) [2,3,4]. The inverse of *Q* is described by the ratio of the damping loss to the stored energy in the resonator, and thus, $Q^{-1}$ is characterized by the energy dissipation in resonators. Clarifying the energy dissipation mechanisms is the key to realizing high-sensitivity mass or force detectors operating at room temperature.

Energy dissipation ($Q^{-1}$) in a NEMS is obtained as a sum of the energy dissipation of each individual loss mechanism, such as support loss, thermoelastic damping and surface loss [5]. When these individual loss mechanisms show different temperature (*T*) dependences, a $Q^{-1}$-*T* curve can characterize the dominant dissipation mechanism. Several studies have reported a specific $Q^{-1}$-*T* curve for doubly-clamped graphene resonators, irrespective of the details of the experimental conditions like the size or actuation scheme of resonators [2,3,4], implying that a certain energy dissipation mechanism commonly exists in doubly-clamped resonators. Recent theoretical studies have pointed out that free edges of graphene resonators could cause the energy dissipation [6,7]. In doubly-clamped graphene resonators, both ends of a graphene sheet are fixed to the substrate, and the other two edges are free. Certain undesired vibrations occur on these two free edges, and these edge vibrations destroy the coherence of the mechanical oscillation. In fact, at room temperature, doubly-clamped resonators with free edges show low *Q* of less than $10^{2}$ [2,3,8,9], while for a circular drumhead resonator, in which all sides are clamped to the substrate, *Q* reaches 2000 [10]. Zande et al. have reported that square graphene membranes clamped on all sides show better device reproducibility and higher *Q* compared with doubly-clamped resonators [3]. These results suggest that eliminating edge vibrations on free edges is important for obtaining high-*Q* graphene resonators. The dissipation properties, such as a $Q^{-1}$-*T* curve, between the resonators with and without free edges have not been compared in experiments so far.

In this article, we review our recent work regarding energy dissipation in our doubly-clamped and circular drumhead graphene resonators [11,12]. Our doubly-clamped trilayer graphene resonator shows a characteristic $Q^{-1}$-*T* curve similar to that seen in monolayer graphene in previous studies: $Q^{-1}$ ∝ $T^{0.3}$ below ∼100 K and ∝ $T^{2}$ above ∼100 K. This indicates the presence of a common energy dissipation mechanism for graphene doubly-clamped resonators, regardless of the layer number. We show that the structure of the resonators plays an important role in discussing this behavior. The characteristic dissipation at low temperatures below 100 K is consistent with the previous numerical studies simulating the edge vibration [6,7]. We can conjecture that the dependence of $Q^{-1}$ at higher temperatures is induced by the effect of tensile strain due to the thermal contraction of the clamped-end metals of the resonators [13]. Furthermore, by comparing $Q^{-1}$-*T* curves obtained for the two types of resonators, with and without free edges, we found that their energy dissipations can be attributed to different mechanisms. We observed that $Q^{-1}$ is proportional to *T* for drumhead resonators in the wide temperature region from 20 K to room temperature, indicating that edge vibrational modes are suppressed. By comparing our results with the numerical studies [6,7,14], we can conclude that *Q* in our drumhead resonators cannot be explained by only the phonon-phonon scattering mechanism, and the difference is attributed to the effects of grain boundaries of graphene. Our experimental results indicate that suppressing the energy dissipation due to edge modes and the effects of grain boundaries are the keys to obtaining high *Q* in graphene resonators.

## 2. Materials and Methods

In this section, we briefly explain how we fabricated our graphene resonators with and without free edges, doubly-clamped and drumhead structures (see [11,12] for details). We also show microscope images to discuss the actual structures of the resonators. The fabrication methods and the resulting resonator structures are important for discussing resonator properties and energy dissipation mechanisms.

### 2.1. Fabrication of Doubly-Clamped Resonators

The doubly-clamped trilayer resonators were fabricated from epitaxial graphene grown on SiC(0001) by photoelectrochemical (PEC) wet etching of SiC [15] combined with hydrogen intercalation [11]. This PEC etching method enables us to precisely design epitaxial graphene resonators including the number of layers. Note that beam thickness is generally a parameter that determines the resonance properties. Graphene on SiC(0001) inevitably has a buffer layer between the SiC substrate and graphene layer. In the buffer layer, carbon atoms are partially bound to silicon atoms of the substrate [16]. To precisely design the graphene NEMS including the layer number, it is desired to elucidate whether the buffer layer is converted to graphene or not after the Si-C bonds have been broken by PEC etching of SiC. Thus, we combined a hydrogen intercalation scheme [17] with the PEC etching process to decouple the Si-C bonds between the buffer layer and the substrate. We used bilayer graphene as a starting material to create suspended trilayer graphene by PEC etching of SiC. First, bilayer graphene was grown epitaxially on an n-type 4H-SiC(0001) substrate by heating the substrate in an ultrahigh vacuum [18]. Doubly-clamped beam structures were patterned on the graphene by photolithography and reactive-ion etching using ${\mathrm{CF}}_{4}$. The beam was patterned into a bow-tie shape. To break Si-C covalent bonds at the interface between a buffer layer and SiC, hydrogen was intercalated by annealing the sample in a hydrogen atmosphere. Au/Cr (200 nm/5 nm) film was deposited as a mask for PEC etching by using electron beam evaporation and lift-off techniques. After these processes, PEC wet etching was performed using the patterned graphene and a Pt wire as the anode and cathode, respectively. Voltage was applied potentiostatically between them with a constant current of 1 mA/${\mathrm{cm}}^{2}$ in an electrolyte of 1 wt % aqueous KOH solution. Xe-UV light was irradiated on the samples to generate electron-hole pairs for the enhancement of the etching reaction.

**Figure 1:**
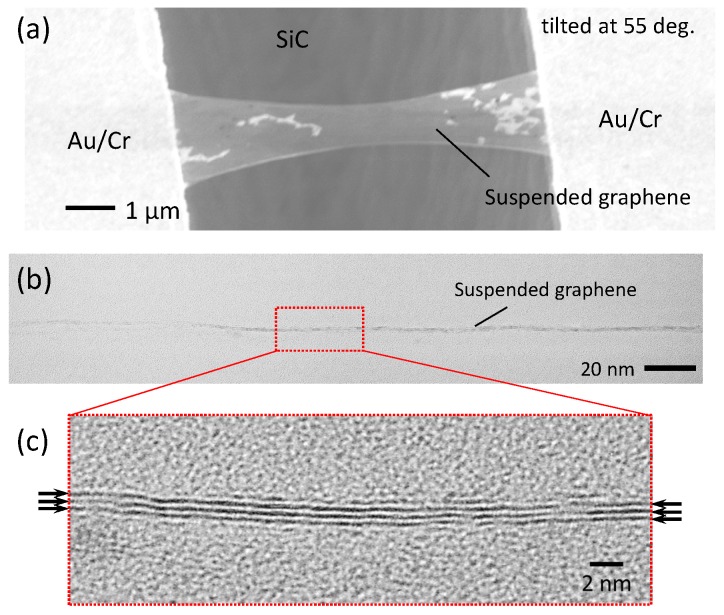
(**a**) Scanning electron microscopy (SEM) image (tilted at 55${}^{\circ }$) of the doubly-clamped suspended graphene. (**b**) Low and (**c**) High magnification cross-sectional TEM images of the suspended graphene. Black arrows in (c) indicate graphene layers. Adapted from [11]. Copyright (2013) The Japan Society of Applied Physics.

Figure 1a shows a scanning electron microscopy (SEM) image taken after the PEC etching had been performed for six hours. We see that the doubly-clamped graphene beam, 7.6 μm in length and 3.7 μm in maximum width, is suspended between the Au/Cr pads. White flakes observed on the graphene are photoresist residues. Figure 1b,c show cross-sectional transmission electron microscopy (TEM) images of the graphene. The low magnification TEM image (Figure 1b) shows that there is no SiC lattice structure under the graphene layer, which means SiC is completely etched. A suspended trilayer structure is clearly observed in Figure 1c, where each black line represents a graphene layer. These TEM images show that the bilayer graphene and buffer layer turned into three graphene layers after PEC etching of the substrate.

### 2.2. Fabrication of Circular Drumhead Resonators

The drumhead resonators were fabricated by growing graphene by the chemical vapor deposition (CVD) method and transferring it to cover circular holes on ${\mathrm{SiO}}_{2}/$Si substrates. First, monolayer graphene was grown on Cu foil at 1000 ${}^{\circ }$C by CVD [19]. To avoid trapping water inside the holes, we performed a dry-transfer method by following procedures described in [20]. A 100-nm thick poly(methyl methacrylate) (PMMA) film was spin-coated on the graphene/Cu foil as a supporting substrate during the transfer process, and it was dried in air. After a 2-mm thick polydimethylsiloxane (PDMS) frame had been stamped on the PMMA/graphene/Cu foil stack, the copper was etched in aqueous solution of 1 mol/L ${\mathrm{FeCl}}_{3}$ and the stack was rinsed in deionized water. The PDMS/PMMA/graphene composite was removed from the water and dried in air, and then, it was placed on the ${\mathrm{SiO}}_{2}/$Si substrate with 200-nm deep holes. To increase the adhesion between graphene and the substrate, the substrate was heated at 170 ${}^{\circ }$C over night. The PDMS frame was peeled off gently; as a result, the PMMA/graphene layer remained on the ${\mathrm{SiO}}_{2}/$Si substrate. Finally, the sample was annealed in Ar of 100 sccm and $H_{2}$ of 100 sccm at 350 ${}^{\circ }$C for two hours to remove the PMMA support layer.

**Figure 2:**
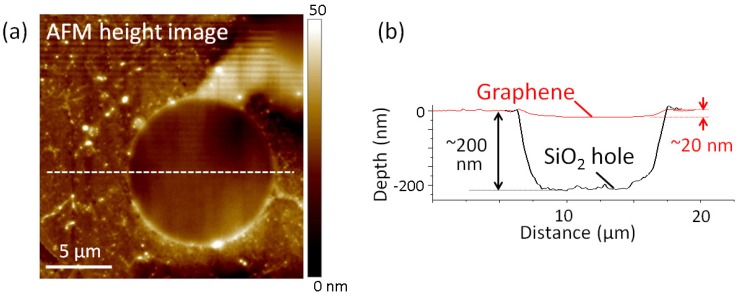
(Color online) (**a**) Atomic force microscopy (AFM) topography of a drumhead graphene resonator. (**b**) Line profile along the dotted line in (a). The red solid line indicates the graphene layer. The black solid line shows the line profile of the hole. Reproduced from [12], with the permission of American Institute of Physics (AIP) Publishing.

The atomic force microscopy (AFM) topographic image in Figure 2 shows that circular graphene is suspended over a hole of 10 μm in diameter. The graphene has a ∼20-nm dip along the edge of the hole. The dip comes from the van der Waals interaction between the edge of the graphene drum and the sidewalls of the hole [21]. For the mechanical oscillation measurement, we used a drumhead resonator with a 20-μm diameter.

## 3. Results and Discussion

### 3.1. Fundamental Resonance Frequency at Room Temperature

**Figure 3:**
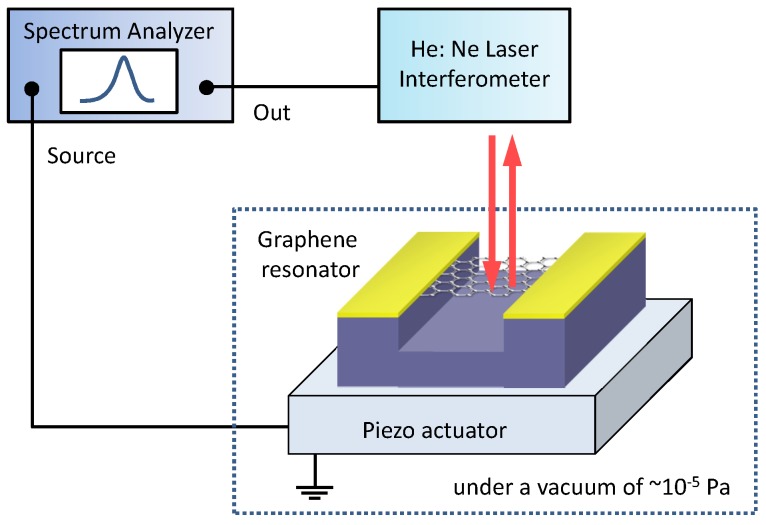
(Color online) A schematic of the detection setup of the mechanical vibration of the resonators.

We first investigated the fundamental resonance frequency $f_{0}$ at room temperature for the graphene resonators with and without free edges with the following method. For this purpose, the vibration of the resonators was induced by applying voltage to a piezo-actuator and detected by using interferometry of the reflected light of a He:Ne continuous-wave laser (633 nm). A schematic image of the detection setup is shown in Figure 3. The resonators were actuated at a low enough driving voltage in the linear amplitude response regime. All measurements were performed under a vacuum of $∼10^{-5}$ Pa. Here, we explore the good modeling of the graphene-resonator structures to reproduce the observed $f_{0}$.

**Figure 4:**
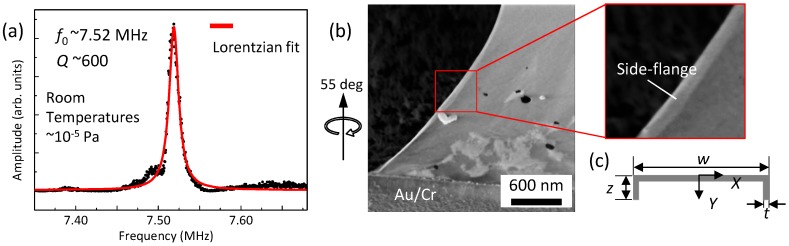
(Color online) (**a**) Amplitude versus frequency for the doubly-clamped trilayer graphene resonator measured at room temperatures under a vacuum of $∼10^{-5}$ Pa. (**b**) A close-up SEM image of the resonator with side-flanges (tilted at 55${}^{\circ }$) and (**c**) a schematic side-view image of the side-flanges. Adapted from [11]. Copyright (2013) The Japan Society of Applied Physics.

Figure 4a shows the amplitude versus frequency of the induced vibration for a doubly-clamped beam of 7.6 μm in length and 3.7 μm in width. From a fit to a Lorentzian, a resonance frequency is evaluated to be $f_{0}$ = 7.52 MHz. The fundamental resonance frequency $f_{0}$ for doubly-clamped resonators under no tension is given by [22]:

(1) $$f_{0}=\frac{4.73^{2}}{2\pi }\frac{1}{L^{2}}\sqrt{\frac{E}{\rho }}\sqrt{\frac{I}{wt}},$$

where the length *L*, width *w* and thickness *t* of our graphene beam are 7.6 μm, 3.7 μm and 1.0 nm, respectively. Given the rectangular shape, we obtain the second moment of area *I* = $wt^{3}/$12 for doubly-clamped beams, leading to $f_{0}$ of 0.37 MHz with Young’s modulus *E* = 1 TPa and mass density ρ = 2200 kg/$m^{3}$ of bulk graphite [23]. Considering the bow-tie shape of the beam with a finite element analysis, we obtain almost the same value of 0.43 MHz. These simple modelings yield ten-times smaller frequencies than the experimentally-observed $f_{0}$ of 7.52 MHz. Higher $f_{0}$ values can be induced by tensile strain [8], whereas Raman measurement revealed that our doubly-clamped resonator has no strain. We comment that Raman G (2D) peaks at 1599 ${\mathrm{cm}}^{-1}$ (2736 ${\mathrm{cm}}^{-1}$) were observed for graphene on SiC before PEC etching, indicating the compressive strain of about 0.5%; this compressive strain was released after PEC etching as a result of a complete etching of the SiC substrate. The Raman measurement also showed a prominent D peak related to defects. However, no G peak broadening or splitting was observed, which means the chemical structure of graphene is maintained. The intensity ratio of Raman D and G peaks is ∼0.3, which suggests that the elastic modulus is nearly unchanged [24]. Thus, the effects of strain and defects on $f_{0}$ are small.

We next discuss the effects of resonator deformation based on the previous study by Shivaraman et al. [15]. They showed that a buckle with a side-flange structure of beams provides a large second moment of area *I* and that the resulting enhancement of the stiffness of the beams causes an increase in $f_{0}$ [15]. In Figure 4b, we found the inhomogeneous deformation at the side of beam. Here, we simply and approximately deal with this deformation as the side-flange structure the same as the previous study. We also simplified the bow-tie-shaped structure. Thus, we consider a simplified beam structure of a rectangular shape with the constant side-flange length *z*, as shown in Figure 4c. Consequently, *I* is rewritten as $I=\left(w-2t\right)t^{3}/12+\left(w-2t\right)t{\left(2y-t\right)}^{2}/4+2\left[tz^{3}/12+tz{\left(2y-z\right)}^{2}/4\right]$ with the position of neutral axis $y=\left[t^{2}\left(w-2t\right)/2+2z^{2}t/2\right]/\left[t\left(w-2t\right)+2zt\right]$ [15]. By using these expressions and Equation (1), the observed $f_{0}$ = 7.52 MHz is reproduced with *z* = 57 nm. Figure 4b provides rough estimation of the length of the side flange ranging from about 30–110 nm with an average value of 60 nm. The calculated side-flange length is consistent with the rough estimation, indicating that the deformation at the edge can be the key to the modeling to reproduce the resonance frequency.

Next, we discuss the fundamental resonance frequency at room temperature for circular drumhead monolayer graphene resonators. For a 20-μm drumhead resonator, the observed fundamental resonance mode is $f_{0}$ = 7.60 MHz. From the peak shift of 2D and G bands in the Raman spectra, we estimate the strain in the drumhead resonator to be about 0.3% [25]. This tensile strain may result from the graphene transfer process, where the van der Waals interaction adheres the graphene drum to the sidewalls of the substrate hole, as shown in Figure 2b. By considering the strain, we obtain a consistent $f_{0}$ value with the observed one as follows. In circular resonators under tension, the fundamental frequency is given by $f_{0}$ = (4.808/2πD)($Y_{t}\epsilon /\rho \alpha {)}^{1/2}$ [22], where *D* is the diameter, $Y_{t}$ is the in-plane Young’s modulus, ϵ is strain, ρ is the in-plane density of graphene and α is the adsorbed mass coefficient. With *D* = 20 μm, $Y_{t}$ = 55 N/m for CVD-graphene [26] and ρ = 7.4 × $10^{-16}$ g$\cdot \mu m^{-2}$, the observed $f_{0}$ = 7.60 MHz is reproduced with ϵ/α = 0.05%. In previous studies [2], α was estimated to be about 6–7, which leads to ϵ = 0.3%–0.4%, consistent with the strain estimated from the Raman spectra. Note that the drumhead shape will be robust to global deformation, while buckling easily occurs in doubly-clamped resonators.

### 3.2. Temperature Dependence of the Inverse of Quality Factors

**Figure 5:**
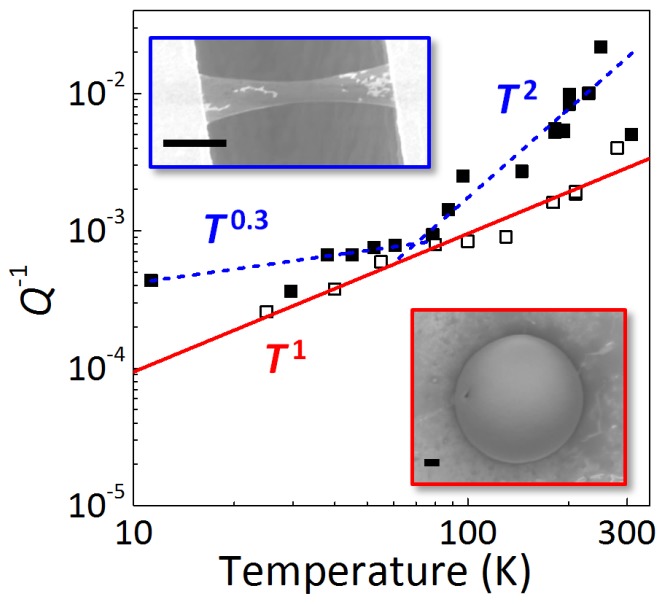
(Color online) Temperature-dependent energy dissipation ($Q^{-1}$-*T*) of our doubly-clamped trilayer graphene resonators (solid squares) and drumhead monolayer graphene resonators (open squares). Blue dashed and red solid lines show linear fittings for doubly-clamped and drumhead graphene resonators, respectively. Insets show SEM images of our trilayer doubly-clamped resonators and monolayer drumhead resonators. Scale bars are 2.5 μm. Reproduced from [12], with the permission of AIP Publishing.

We next discuss what limits *Q*. The obtained *Q* may include the effects of both energy dissipation and dephasing [27]. Our resonators were actuated at a low enough driving voltage in the linear amplitude response regime, so that the effects of dephasing would be small [27]. In the following, we thus focus on the energy dissipation as the dominant source of decreases in *Q*. To determine what the dominant energy dissipation mechanism is, we compare the temperature dependence of $Q^{-1}$ between the resonators with and without free edges. Figure 5 shows the temperature *T* dependence of $Q^{-1}$ of fundamental modes for our doubly-clamped trilayer graphene resonator and drumhead monolayer graphene resonator. The two resonators show the different temperature dependence of energy dissipation. The doubly-clamped resonator shows the characteristic dissipation depending on temperature: $Q^{-1}$ rapidly decreases with ∝ $T^{2}$ and then decreases more slowly with ∝ $T^{0.3}$ below 80 K. On the other hand, the drumhead resonator shows $Q^{-1}$ proportional to *T*. We found that the drumhead resonators have higher *Q* in wide temperature ranges, because $Q^{-1}$-*T* curves have a higher (lower) power index at low (high) temperatures.

**Figure 6:**
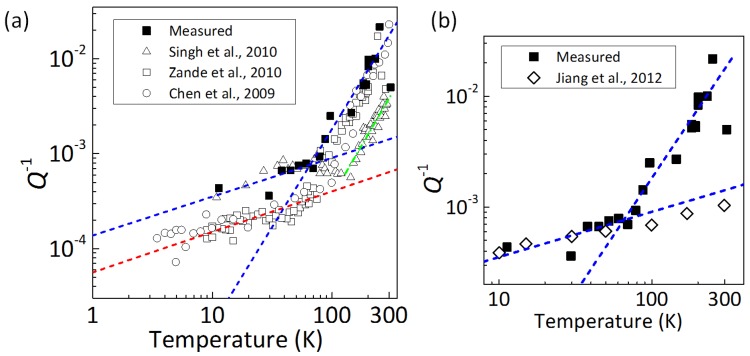
(Color online) (**a**) Temperature-dependent energy dissipation ($Q^{-1}$-*T*) of doubly-clamped graphene resonators for our trilayer graphene resonators (solid squares) and monolayer graphene resonators in [2] (open circles), [3] (open squares) and [4] (open triangles). Blue dashed lines show linear fittings at different temperature ranges for our trilayer graphene resonators. The red dashed line indicates a linear fitting for monolayer graphene resonators in the lower temperature region. The green dashed line indicates a linear fitting for monolayer graphene resonators in [4] in the higher temperature region. (**b**) Comparison of $Q^{-1}$-*T* curves between our doubly-clamped trilayer resonators and the molecular dynamics simulation result in [7] (open rhombuses). Reproduced from [12], with the permission of AIP Publishing.

We begin by discussing what the dominant energy dissipation mechanism is in doubly-clamped resonators. In Figure 6a, we show the $Q^{-1}$-*T* curves for doubly-clamped resonators reported in the following previous papers, namely for monolayer graphene resonators fabricated by mechanical exfoliation [2,4] and by CVD [3], where their vibration was detected by electrical readout. All of the data show a qualitatively similar temperature dependence of $Q^{-1}$ to $T^{0.3-0.4}$ below ∼100 K and $T^{2}$ when 100 <T< 300 K. Interestingly, the temperature dependence is observed irrespective of the details of the samples and measurement methods. This means that the dissipation mechanism is a general feature of doubly-clamped graphene resonators.

One of the candidates of the common dissipation mechanisms in the doubly-clamped resonators is the spurious edge mode that occurs at the free edges of the beams as discussed in [6,7]. Molecular dynamics (MD) simulations showed that free edges flip during the mechanical oscillation of graphene resonators, and this flip motion breaks the coherence of the mechanical oscillation [6,7]. As a result, the quality factor *Q* decreases with a temperature dependence of $Q^{-1}$ ∝ $T^{0.28}$, which is shown as open rhombuses in Figure 6b. Our results showing $T^{0.3}$ are quantitatively consistent with the numerical results. This indicates that energy dissipation caused by edge vibrations localized at free edges is dominant below about 80 K, and this feature is common for doubly-clamped resonators and independent of other sample details, such as size and the layer number. This layer number-independent feature suggests that energy dissipation mechanisms unique to multilayer graphene, such as interlayer frictional losses due to weak van der Waals interactions [28], are small. Meanwhile, the temperatures at which the power index changes range from 50 to ∼100 K, depending on the samples: 50 K [2,3] and ∼100 K [4] for monolayer graphene; 80 K for our trilayer graphene resonator. Figure 6a also shows that our sample (solid squares) and the samples in [4] (open triangles) have a rather low *Q* in the low-temperature range.

We next discuss the qualitative difference in *Q* of the doubly-clamped resonators in the low-temperature region. It has been suggested that the deformation of the beams will decrease *Q*. Sanchez et al. have clarified by scanning probe microscopy that the local deformation at the edges can increase the possibility of the stochastic appearance of the edge vibration modes [9]. Figure 4b shows that our resonators have side flanges on their free edges. Such side-flanges increase the probability of edge modes, leading to low *Q*.

We further discuss the energy dissipation source at higher temperatures, i.e., the $T^{2}$ tendency above ∼100 K in doubly-clamped graphene resonators. A very intrinsic dissipation mechanism, phonon-phonon scattering, obeys a linear *T* dependence of $Q^{-1}$ [7,29] and, thus, cannot explain $T^{2}$ behavior. The adsorbate migration mechanism can explain a change in the slope of the $Q^{-1}$-*T* curves, which has been discussed by Jiang et al. with MD simulations [30]. Such a $Q^{-1}$-*T* slope strongly depends on the density and mass of the adsorbates on the graphene resonator surface, which is inconsistent with the experimentally-observed sample-independent feature. Another possible origin is thermoelastic damping: namely, the temperature gradient that depends on the portion of the beam causes a large energy dissipation. We expect that the side-flange structure of our resonators induces a large temperature difference. We used the finite element method to calculate the thermoelastic energy loss in our resonator. On the basis of the results, as expected, we found that the side-flange in our resonator induces a large temperature difference, depending on the portion of the resonator, which reduces *Q* with increasing flange length. However, even for the side-flange length of 60 nm observed by SEM, *Q* at room temperature is estimated to be ∼$10^{4}$, which is two orders of magnitude higher than the measured values. The $Q^{-1}$ calculated based on the thermoelastic damping follows $T^{1.4}$ from 160 down to 10 K, which rather differs from the experimental results of $T^{2}$. We finally discuss another candidate of the dominant dissipation mechanisms, which is tensile strain induced by the clamping metal. As temperature decreases, the Au metal will induce tensile strain in graphene due to thermal contraction. On the other hand, the contribution of ${\mathrm{SiO}}_{2}/$Si substrates used in [2,3,4] is very small [4]. In our samples, the substrate effects are small, because the thermal expansion coefficient of SiC is an order of magnitude smaller than that of Au. It has been reported that applying tensile strain increases *Q* both experimentally [31,32] and theoretically [6,14]. The thermal expansion coefficient of Au drops to zero below about 100 K, and then, the effect of the tensile strain will be small [13,33]. A characteristic temperature of about 100 K at which the power law changes can be reasonably explained by this dissipation mechanism. Note that the MD simulation shown as open rhombuses in Figure 6b does not deal with strain caused by the clamping metal. In addition, we found experimentally that drumhead resonators without clamping metal show no change in the temperature dependence of $Q^{-1}$ from over 100 K up to room temperature. Thus, the temperature dependence of the tensile strain induced by the clamping metal is expected to be dominant above 100 K.

**Figure 7:**
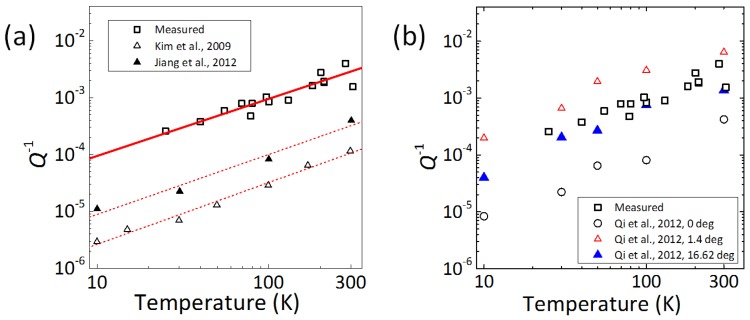
(Color online) (**a**) Energy dissipation ($Q^{-1}$) as a function of temperature in drumhead monolayer graphene resonators. Our measured data are shown as open squares. Solid triangles and open triangles are the quality factors estimated by the numerical analysis for the circular resonator with constrained edges from [6] and for the rectangular-shaped resonator with the periodic boundary condition from [7], respectively. Red solid and dashed lines show linear fittings. (**b**) Variation of energy dissipation ($Q^{-1}$) as a function of temperature in drumhead monolayer graphene resonators. Our measured data (open squares) are compared with calculated data in [14] for the resonators under 1% strain without grain boundaries (open circles) and with a single grain boundary with misorientation angles of 1.4${}^{\circ }$ (red open triangles) and 16.62${}^{\circ }$ (blue solid triangles). Reproduced from [12], with the permission of AIP Publishing.

We move our focus to drumhead graphene resonators and discuss the dominant source of energy dissipation in resonators without free edges. Figure 7a shows the *T*-dependence of $Q^{-1}$ for our experimental data and MD simulations for resonators without free edges from [6,7]. The MD simulations calculated *Q* of monolayer graphene for a circular resonator with constrained edges in [6] and for a rectangular resonator with the periodic boundary condition in [7]. These numerical results show the temperature scaling of $Q^{-1}$ ∝ *T*, which is in good agreement with our experimental data. This behavior can be explained by the intrinsic phonon-phonon scattering [29]. This means that the edge-induced dissipation in graphene resonators is eliminated by securing all edges, and consequently, the intrinsic phonon-phonon scattering becomes dominant in drumhead resonators. We should note that, since our drumhead resonator has no metal pads, the energy dissipation with $Q^{-1}\propto T^{2}$ cannot be observed even at around room temperature.

We further discuss the qualitative difference between the measured $Q^{-1}$ and the calculated ones shown in Figure 7a. Qi et al. have shown that the grain boundaries of graphene affect *Q* [14], and in Figure 7b, we compare our $Q^{-1}$ vs. *T* data with those calculated in [14] by MD simulation by considering a single grain boundary with misorientation angles. We estimate the size of a grain in our CVD graphene to be several micrometers, which is smaller than our resonators, which have a diameter of about 20 μm. We therefore expect that the effects of grain boundaries in our samples can be reasonably explained by the simulations with a single grain boundary [14]. In fact, our experimental results quantitatively agree with the MD simulation results for graphene under 1% strain by Qi et al. Note that our drumhead resonator is under a strain of ∼0.3% estimated from Raman G and 2D peak positions. The MD simulation indicates that grain boundaries degrade *Q* in drumhead graphene resonators and that $Q^{-1}$ is proportional to $T^{1.1}$ [14]. This power index is almost the same as that of the intrinsic phonon-phonon scattering, and thus, $Q^{-1}$ ∝ *T* will be the minimal energy dissipation. We should note that the grain boundary effects are negligible in the doubly-clamped resonators made from CVD graphene [3], because the edge vibration effects are much more dominant. Our experimental results demonstrate that the $Q^{-1}$ ∝ *T* dependence of our drumhead resonators in a wide temperature range leads to higher *Q* than that of our doubly-clamped resonators. We can thus stress that minimizing the energy dissipation allows us to create high-*Q* graphene resonators. If we can remove grain boundaries in drumhead resonators, for example by using CVD graphene with 100-μm grains [34,35], or millimeter grains [36,37,38], or by using epitaxial graphene on SiC [39], we should be able to obtain high-*Q* graphene resonators.

We finally comment that, in the very low temperature region of 20 mK <T< 1 K, a doubly-clamped carbon-nanotube resonator shows $Q^{-1}\propto T^{0.3-0.4}$ even though a nanotube has no free edges [40]. This dissipation might be due to a local mode caused by defects in distorted lattices [41]. These might imply that the $T^{0.3-0.4}$ dependence is common for nanoscale resonators with free edges or defects in lattices, and the drumhead resonators might show $T^{0.3-0.4}$ behavior at very low temperatures. We further comment that the interlayer friction causes additional energy dissipation in drumhead multilayer graphene resonators, while the present drumhead monolayer graphene resonators are expected to be affected mainly by intrinsic phonon-phonon scattering. Numerical simulation has shown that the drumhead double layer graphene resonator shows $Q^{-1}$ proportional to $T^{0.32}$ with lower *Q* values than the monolayer one due to the interlayer friction [42].

## 4. Conclusions

Energy dissipation mechanisms in graphene resonators with and without free edges were discussed by examining the $Q^{-1}$-*T* curves. A specific $Q^{-1}$-*T* curve observed in doubly-clamped graphene resonators indicates that there is a common energy dissipation mechanism: $T^{0.3}$ dependence at low temperatures is caused by vibrations at the free edges of the beam. $T^{2}$ dependence is due to the tensile strain induced by clamping Au metals. On the other hand, drumhead resonators show linear-*T* behavior of $Q^{-1}$ down to the lower temperatures, which is due to the intrinsic phonon-phonon scattering. Because of the higher power law at low temperatures, the drumhead resonators have higher *Q* than doubly-clamped resonators. This suggests that removing the free edges may allow us to reach high *Q*. The grain boundaries of graphene in the drumhead resonators also decrease *Q*, and such imperfections will generally be an obstacle to high-*Q* resonators. The recent achievement of the CVD-growth of millimeter-scale graphene domains and the development of dry-transfer methods are promising for creating graphene-based NEMS without imperfections, which will provide high *Q*.
